# Dataset of driving behaviours in Selangor, Malaysia

**DOI:** 10.1016/j.dib.2020.105783

**Published:** 2020-05-29

**Authors:** Huay Woon You, Amirah Abdul Rahman, Lutfil Hadi Hendri Dwisatrya

**Affiliations:** Pusat GENIUS@Pintar Negara, Universiti Kebangsaan Malaysia, 43600 UKM Bangi, Selangor, Malaysia

**Keywords:** Driving behaviour, Improper overtaking, Tail-gating, Disobey traffic light, Speeding, Mobile phone use while driving

## Abstract

Road traffic accidents have been recognised as a leading cause of death, and one of the prominent public health problems. The human factor, which is the driving behaviour in particular, is said to be the main cause. In line with this, the objective of this research is to present a data article on the response of driving behaviours among drivers. Driving behaviours have been classified into five dimensions, which are speeding, improper overtaking, mobile phone use while driving, tail-gating and disobeying traffic lights. A quantitative study was conducted with a sample size of 160 drivers consisting of residents in a suburban of Selangor, Malaysia. A stratified random sampling method was adopted to identify the respondents. Data analysis was presented in the form of descriptive statistics and tables. The findings show that the majority of respondents agreed that they have driving behaviours that involve improper overtaking, tail-gating and disobeying traffic lights.

Specifications table

**Table:** 

Subject	Human Factors and Ergonomics
Specific subject area	Driving Behaviours
Type of data	Table Numeric Text
How data were acquired	Survey
Data format	Raw Analysed Descriptive
Parameters for data collection	The data was collected from residents in a suburban who have a driving license.
Description of data collection	The respondents were selected based on stratified random sampling. A survey design using a questionnaire was employed in data collection. A reliability test was applied before data analysis. The questionnaire is provided as a supplementary file.
Data source location	Selangor, Malaysia
Data accessibility	With the article

## Value of the data

• The data will be useful to analyse and provide details on the responses of driving behaviours among drivers.
• The data is valuable to identify a strategic program to promote overall road safety.
• The data can be used by policy makers to better understand the importance of driving behaviours in road safety, which is useful for future research.

## 1 Data Description

The survey was conducted using a questionnaire to collect data about driving behaviour among drivers. The questionnaire is provided as a supplementary file. Table 1 presents the demographic characteristics for the samples. Out of the 160 respondents, 86 (53.8%) were female. According to [1], driving experience is one of the crucial factors that may influence the frequency of transport accident involvement of a driver.

**Table 1:** Demographic characteristics of the respondents.

	Frequency	Percent (%)
**Gender**		
Male	74	46.3
Female	86	53.8
**Driving experience**		
Less than 1 year	17	10.6
1 – 2 years	27	16.9
3 – 4 years	25	15.6
5 – 9 years	30	18.8
10 -14 years	15	9.4
15 years and above	46	28.8
**Previous involvement in road accidents**		
Never	53	33.1
1 – 2 times	71	44.4
3 – 4 times	24	15.0
5 – 6 times	8	5.0
More than 6 times	4	2.5

Here, 46 respondents had a driving experience of 15 years and above (28.8%), while 30 respondents had a driving experience of 5-9 years (18.8%). Only 15 respondents have a driving experience of 10-14 years (9.4%), and 17 have less than 1 year of driving experience (10.6%). Moreover, more than half of the respondents (91) have over 5 years of driving experience (see Table 1).

Table 1 demonstrates that 107 (66.88%) of the respondents have been involved in at least one transport accident while driving a vehicle. Driving behaviours of the driver may influence the frequency of traffic accident involvement. Drivers who are involved in a lot of traffic accidents might have inappropriate driving behaviours [2, 3]. Driving behaviours have been divided into five different categories, i.e. speeding, improper overtaking, mobile phone use while driving, tail-gating and disobeying traffic lights [4].

Table 2 shows that 111 respondents were reported to have improper overtaking driving behaviour; this represents the highest driving behaviour. Next, tail-gating has been identified as the second highest driving behaviour, which was mentioned by 105 respondents. A total of 96 respondents confessed that they disobey traffic lights while driving. Among the five dimensions of driving behaviours, only mobile phone use while driving and speeding was answered by almost half of the respondents, which are 85 and 82 respondents, respectively.

**Table 2:** Classification of respondent by driving behaviours.

Driving Behaviour	Frequency	Percent (%)
Speeding		
Yes	82	51.3
No	78	48.8
Improper Overtaking		
Yes	111	69.4
No	49	30.6
Mobile Phone Use while Driving		
Yes	85	53.1
No	75	46.9
Tail-gating		
Yes	105	65.6
No	55	34.4
Disobey Traffic Light		
Yes	96	60
No	64	40

Table 3 displays the distribution of respondents’ driving behaviours by driving experiences. In general, it is observed that the respondents with more years of driving experience will have more appropriate driving behaviours compared to respondents with less years of driving experience. For example, driving behaviour of tail-gating was answered by 88.2% of the respondents with less than 1 year of driving experience, compared to 67.4% of the respondents with 15 years and above driving experiences.

**Table 3:** Distribution of respondent driving behaviours by driving experiences.

Driving Behaviour	Driving Experience (n %)					
	Less than 1 year	1 – 2 years	3 – 4 years	5 – 9 years	10 – 14 years	15 years and above
Speeding						
Yes	12 (70.6)	11 (40.7)	11 (44)	17 (56.7)	5 (33.3)	26 (56.5)
No	5 (29.4)	16 (59.3)	14 (56)	13 (43.3)	10 (66.7)	20 (43.5)
Improper Overtaking						
Yes	15 (88.2)	20 (74.1)	14 (56)	21 (70)	11 (73.3)	30 (65.2)
No	2 (11.8)	7 (25.9)	11 (44)	9 (30)	4 (26.7)	16 (34.8)
Mobile Phone Use while Driving						
Yes	12 (70.6)	13 (48.1)	9 (36)	14 (46.7)	8 (53.3)	29 (63.0)
No	5 (29.4)	14 (51.9)	16 (64)	16 (53.3)	7 (46.7)	17 (37)
Tail-gating						
Yes	15 (88.2)	16 (59.3)	17 (68)	15 (50)	11 (73.3)	31 (67.4)
No	2 (11.8)	11 (40.7)	8 (32)	15 (50)	4 (26.7)	15 (32.6)
Disobey Traffic Light						
Yes	13 (76.5)	19 (70.4)	13 (52)	12 (40)	10 (66.7)	29 (63.0)
No	4 (23.5)	8 (29.6)	12 (48)	18 (60)	5 (33.3)	17 (37)

Furthermore, Table 4 indicates that the involvement in road accidents may have been influenced by driving behaviour. For the respondents who were involved in road accidents at least once, at least 50% of the respondents had inappropriate driving behaviours. Nevertheless, there is an exception for driving behaviours, such as mobile phone use while driving (3-4 times and more than 6 times), disobeying traffic lights (3-4 times and more than 6 times) and tail-gating (5-6 times). In addition, 75% of the respondents with more than 6 times of involvement in road accidents have driving behaviours in speeding, improper overtaking and tail-gating. These driving behaviours are similar to the responses with high frequency provided by the respondents (see Table 2).

**Table 4:** Distribution of respondent driving behaviours by previous involvement in road accidents.

Driving Behaviour	Previous Involvement in Road Accidents				
	Never	1 – 2 times	3 – 4 times	5 – 6 times	More than 6 times
Speeding					
Yes	27 (50.9)	36 (50.7)	12 (50)	4 (50)	3 (75)
No	26 (49.1)	35 (49.3)	12 (50)	4 (50)	1 (25)
Improper Overtaking					
Yes	39 (73.6)	50 (70.4)	13 (54.2)	6 (75)	3 (75)
No	14 (26.4)	21 (29.6)	11 (45.8)	2 (25)	1 (25)
Mobile Phone Use while Driving					
Yes	29 (54.7)	43 (60.6)	8 (33.3)	4 (50)	1 (25)
No	24 (45.3)	28 (39.4)	16 (66.7)	4 (50)	3 (75)
Tail-gating					
Yes	37 (69.8)	48 (67.6)	14 (58.3)	3 (37.5)	3 (75)
No	16 (30.2)	23 (32.4)	10 (41.7)	5 (62.5)	1 (25)
Disobey Traffic Light					
Yes	35 (66)	47 (66.2)	10 (41.7)	4 (50)	0 (0)
No	18 (34)	24 (33.8)	14 (58.3)	4 (50)	4 (100)

## 2 Experimental Design, Materials, and Methods

A cross sectional quantitative study was conducted in a suburban of Selangor, Malaysia. An informed consent was obtained from each respondent prior to this study. The participation of the respondents was entirely voluntary, and that the respondent has the right to decline to participate, without consequence, at any time. Moreover, the participation of the respondent is anonymous. This study was done among 160 drivers. Data were collected by means of a structured questionnaire, consisting of three sections. Demographic information of the respondents was obtained using Section 1. Section 2 identified the driving experience and previous involvement in road accidents. Section 3 assessed the driving behaviours. Descriptive statistics was utilized in data analysis and presented in Tables 1 to 4.

## Declaration of Competing Interest

The authors declare that they have no known competing financial interests or personal relationships which have, or could be perceived to have, influenced the work reported in this article.

## Appendix Supplementary materials

Image, application 1

Image, application 1

Image, application 1

Image, application 1

Image, application 1

Image, application 1
