# Supplementary material for: Dataset of driving behaviours in Selangor, Malaysia
Source: Data Brief. 2020 May 29;31:105783. doi: 10.1016/j.dib.2020.105783 (PMC7334400; doi:10.1016/j.dib.2020.105783)
Supplement: Supplementary file 1 [file mmc1.pdf]

CRediT (Contributor Roles Taxonomy) author statement

You Huay Woon: Conceptualization, Methodology, Validation, Formal analysis, Writing – Original draft, Writing -Review & Editing, Supervision **Amirah Abdul Rahman:** Conceptualization, Methodology, Validation, Formal analysis, Investigation, Writing – Original draft, **Lutfil Hadi Hendri Dwisatrya:** Conceptualization, Methodology, Validation, Formal analysis, Investigation, Writing – Original draft,
