# Supplementary material for: Dataset of driving behaviours in Selangor, Malaysia
Source: Data Brief. 2020 May 29;31:105783. doi: 10.1016/j.dib.2020.105783 (PMC7334400; doi:10.1016/j.dib.2020.105783)
Supplement: Supplementary file 6 [file mmc6.pdf]

Please answer all the questions from Section A to Section C. All of the information given will be kept confidential.

Section A – DEMOGRAPHIC

1. Gender ( ) Male ( ) Female

Section B – DRIVING EXPERIENCE

2. Driving Experience

   ( ) less than 1 year ( ) 1 – 2 years ( ) 3 – 4 years

   ( ) 5 – 9 years ( ) 10 – 14 years ( ) 15 years and above

3. Experience in road accident involvement

   ( ) never ( ) 1 – 2 times ( ) 3 – 4 times

   ( ) 5 – 6 times ( ) more than 6 times

Section C – DRIVER'S BEHAVIOR ON THE ROAD

Please CIRCLE your answer on the choices provided.

1. I drive over the allowed speed limit YES NO
2. I overtake a vehicle in an area that is prohibited on overtaking YES NO
3. I use a mobile phone without headset when driving YES NO
4. I read and reply SMS when driving YES NO
5. I disobey the traffic rules when I am running out of time YES NO
6. I drive closely to the vehicle in front of me with the purpose of urging the driver YES NO
7. I use the left shoulder path to overtake a vehicle during traffic jams YES NO

THANK YOU FOR YOUR COOPERATION
